# Expression of both N- and C-terminal GFP tagged huCD36 and their discrepancy in OxLDL and pRBC binding on CHO cells

**DOI:** 10.1186/1476-511X-6-24

**Published:** 2007-09-21

**Authors:** Jianshe Zhang, Ian Crandall

**Affiliations:** 1 Department of Bioengineering and Environmental Science, Changsha University, Changsha, Hunan, China 41003; 2 Department of Medicine, University of Toronto, Toronto, ON, Canada M5S 1X8

## Abstract

CD36, an 88 kDa membrane glycoprotein, is found in several cell types and it has been characterized to have two hydrophobic domains at their N- and C-termini which are essential for protein folding and targeting. In this study, we first tagged the green fluorescent protein (GFP) to both the N- and C-termini of huCD36 and investigated their cellular expression and influences on lipoprotein and plasmodium falciparium parasitized erythrocytes (pRBC) binding. Our work revealed that huCD36 proteins are expressed normally irrespective of the GFP tag presence at either the N- or C-termini. However, the two recombinant proteins showed discrepancy in uptake and surface-binding of OxLDL but they did not affect pRBC binding. These results suggested that the interaction between oxLDL and CD36 could be blocked using recombinant proteins and this may be useful in potential control of the trafficking of modified lipoproteins into monocytes leading to atherogenesis.

## Background

Receptor-mediated binding and uptake of oxidized low lipoprotein (OxLDL) by microphages has been implicated in foam cell formation in the process of atherosclerosis [1,2], in which oxidation of LDL is a critical early event in the pathogenesis and OxLDL is the primal source of lipid that accumulates within cells of the atherosclerotic lesion [1,3,4]. There are at least two major classes of mammalian monocyte and microphage scavenger receptors, termed as SR-A and SR-B, and they are involved in binding/uptake of oxLDL to macrophages and endothelial cells leading to atherosclerotic pathogenesis, and they also function in the recognition, and clearance of damaged tissues or apoptotic cells [5-7].

CD36, an 88 kDa membrane glycoprotein, is found in several cell types, such as platelets, monocytes, macrophages and endothelial cells [8-12]. CD36 has been reported to be a multifunctional receptor and it recognizes a wide variety of ligands including OxLDL [13], thrombospondin [14,15], collagen [16,17], apoptotic neutrophils [18,19], *Plasmodium falciparum*-infected erythrocytes [20-22] and anionic phospholipids [23,24]. Further studies demonstrated that CD36 expressed in COS 7 or Sf9 cells functioned as a high-affinity receptor not only for OxLDL, but also for HDL, LDL and VLDL [10,25]. Several regions of CD36 have been implicated as binding domains for its different ligands, including amino acids 28–93 as the OxLDL binding domain [26], and amino acids 93–120 as the thrombospondin binding region [27].

CD36 protein has been characterized to have two hydrophobic domains at their N- and C-termini and these domains are essential for protein folding and targeting [28]. However, Pucent Navazo et al (1996) suggested that only the transmembrane domain and the C-terminal end of CD36 function in membrane anchoring [29]. Even though different ligand-binding domains on CD36 molecules have been characterized, there specific functions still remain inconclusive. To this end, several attempts were made to define domains for specific ligands and their functions on CD36. Pearce et al. (1998) used a series of GST/CD36 fusion proteins to define the domains of CD36 that specifically bind to Ox-LDL [26]. Stewart and Nagarajan (2006) created a soluble CD36 cDNA by replacing a signal peptide sequence of a type I membrane protein, CD59, at the N-terminus and a human IgG1 CVH2-CH3 Fc domain at the C-terminus of CD36. Their results confirmed that the chimeric sCD36-Ig is secreted and folded correctly, and it competitively inhibits oxLDL binding to membrane-expressed CD36 and oxLDL-induced monocyte adhesion [30,31]. In other studies, different ligand-binding domains of CD36 molecules were detected immunologically with a large group of antibodies raised against CD36 [29,32-34].

In this study, we first tagged the green fluorescent protein to both the N- and C-termini of huCD36 and investigated their cellular expression and influences on lipoprotein and pRBC binding. Our work revealed that huCD36 proteins are expressed normally irrespective of the GFP tag presence at either the N- or C-termini. However, the two recombinant proteins showed discrepancy in uptake and surface-binding of OxLDL but they did not affect pRBC binding. These results suggested that the interaction between oxLDL and CD36 can be blocked using recombinant proteins and this may be useful in potential control of the trafficking of modified lipoproteins into monocytes leading to atherogenesis.

## Methods

### Chemicals and materials

pEGFP-C3 and pEGFP-N3 plasmid DNA were purchased from Clontech Laboratories, Inc. (Pulo Alto, CA, USA). TheExgen 500 transfection kit and restriction enzymes, KphI and HindIII were from Fermentas (Burlinton, ON, Canada). Taq DNA polymerase and nucleotide mix were from Invitrogen Canada (Burlington, ON). Plasmid DNA purification kits were from Qiagen. Anti-CD36 antibody was from Immunotech, Bekiman Coulter (USA). CHO cell lines and stable transfected CHO-CD36 cells were cultured and maintained as described [20].

### Construction of pEGFP-C3-CD36 and pEGFP-N3-CD36

The vectors, pEGFP-C3 and pEGFP-N3, were cut at cloning sites with restriction enzymes, KpnI and Hind III and then purified with Qiagen DNA purification kit. The coding sequence of huCD36 was PCR amplified from pCDNA3-huCD36. For the pEGFP-C3-CD36 construct, two primers were used as 5' ATTAAGCTTATGGGCTGTGACCGGAACTGTG and 5' ATTGGTACCTTATTTTATTGTTTTCGATCTGCA. While for construction of pEGFP-N3-CD36, a stop codon at C-terminal of CD36 was deleted by using the backward primer as 5' ATTGGTACCTTTTATTGTTTTCGATCTGCA. For both PCR-amplified CD36 products, a Hind III site was created at N-terminal and a Kpn I site was created at the C-terminus. Both PCR-CD36 products were restriction-enzyme cut with Hind III and Kpn I and gel-purified. The purified PCR-CD36 was inserted into either peGFP-C3 or pEGFP-N3 vectors by normal ligation procedures. Ligated pEGFP-C3-CD36 and pEGFP-N3-CD36 were transformed into Xl-1Blue competent cells and amplified. Recombinant DNAs were sequenced to determine whether the CD36 coding sequences were inserted correctly into the vectors.

### Transfection and recombinant protein expression assays

CHO cells were seeded on coverslips and cultured in 12-well plates in RPM1640 medium overnight to allow cells to grow to about 70% confluence at the time of transfection. The recombinant plasmid DNAs, either pEGFP-C3-CD36 or pEGFP-N3-CD36, were highly purified to the OD260/OD280 ratio of at least 1.8 or larger. For transfection, ExGen 500 in vitro transfection kit (Invitrogen) was used according to the manufacturer's instructions. Briefly, 4 μg DNA of pEGFP-C3-Cd36 or pEGFP-N3-CD36, were diluted in 50 μl of 150 mM NaCl mixed with 50 μl of diluted Exgen 500 solution (1: 6 with 150 mM NaCl), and incubated for 10 min, and then the mixtures were added to each well with 0.9 ml of fresh RPM1640 medium. The transfected cells were cultured for 48 hours. Transfected fusion gene expression was assayed either with flow cytometery or confocal microscopy.

### Assays of dil-oxLDL binding

After transfection for 48 hours, cells were assayed for oxLDL binding. For uptaking assays, 1 ml of fresh RPM1640 medium was replaced in each well and a designated amount of Dil-oxLDL was directly added to each well and remained in culture at 37°C for 2 – 4 hours. Cells were then washed twice with PBS and fixed with 4% formaldehyde in PBS for 15 min. After washing several times with PBS, cells were mounted onto slides. For surface binding assay, cells after 48 h post-trasnfection were fixed with 4% formaldehyde for 20 min. Upon washing three times with PBS, cells were incubated with Dil-oxLDL at 10 μg/ml in PBS buffer at room temperature for 4 hours. After washing three times, the amount of bounded dil-oxLDL was assayed by flow cytometery or confocal microscopy.

### Immunofluorecent staining of transfected cells with anti-CD36 antibody

To assay if recombinant CD36 could be normally expressed, cells after transfection with either pEGFP-C3-CD36 or pEGFP-N3-CD36 for 48 hours, were immnostained with anti-CD36 antibody at 1:100 in blocking buffer (1% BSA and 0.1% Tween-20 in PBS) at room temperature for 4 hours, or at 4°C overnight. Immediately after primary antibody incubation, cells were washed three times and counterstained with TRIC-conjugated anti-mouse IgG secondary antibody at 1:500 for two hours. After washing three times with PBS, stained cells were assayed by either flow cytometery or confocal microscopy.

### pRBC adhesion assays

Adhesion assays were carried out as described by Crandall et al (1999) [20]. Transfected cells were fixed in 4% formaldehyde in PBS for 15 min and then rinsed three times with BTS. Adhension assays using a total volume of 300 μl of infected blood were carried out. The erythrocytes were kept in suspension by continuous agitation and incubated with the target cells for 90 min. Adhesion was quantitated by direct microscopic observation of transfected cells of either pEGFP-C3-CD36 or pEGFP-N3-CD36. Cells transfected with only GFP were used as control. Three independent experiments were done and data points represent the mean values with the standard error of the mean indicated by the mean bars.

## Results

### Construction and expression of hCD36-GFP fusion proteins in CHO cells

**Figure 1:**
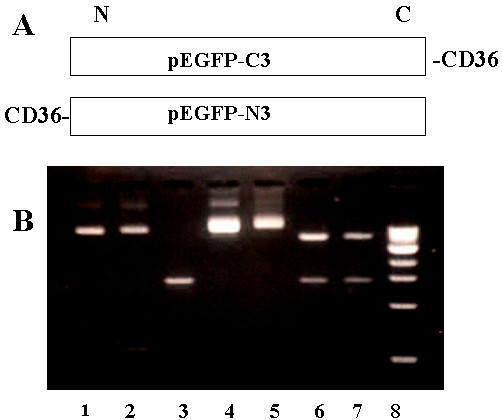
**Schematic diagram of p-EGFP-C3-CD36 and peGFP-N3-Cd36 constructs**. A, the coding sites of vectors, pEGFP-C3 and pEGFP-N3, were restriction enzymatically cut with both Hind III and Kpn I and the DNA fragments were purified. The inserts, PCR-amplified hCD36 coding sequences after purification, were ligated into the vectors, resulted in recombinant constructs of pEGFP-C3-CD36 or pEGFP-N3-CD36. B, 2% agarose gel to confirm the DNA constructs. Lane 1 and 2, Hind III and Kpn I cut and then purified pEGFP-C3 and pEGFP-N3 plasmid DNAs; Lane 3, purified PCR-amplified huCD36 cDNAs; Lane 4, PEGFP-C3-huCD36 constructs; Lane 5, pEGFP-N3-huCD36 constructs; Lane 6 and 7 are re-digested pEGF-C3-hCD36 and pEGFP-N3-hCD36 with Hind III and Kpn I. Lane 8, 1 Kb DNA markers.

The scavenger receptor CD36 is a 78 – 88 kDa plasma membrane glycoprotein and acts as a receptor for OxLDL and acetylated LDL in vitro [10,35,36]. The coding sequences of hCD36 from base 211 to base 1630 (1439 bp) were PCR-amplified and inserted into both pEGFP-C3 and pEGFP-N3 multiple cloning sites at Hind III and Kpn I (Fig. 1A). The recombinant DNAs were twice confirmed by DNA sequencing and restriction enzyme re-cut (Fig. 1B) and the results proved that the coding sequences of hCD36 were inserted in frame in both plasmids.

**Figure 2:**
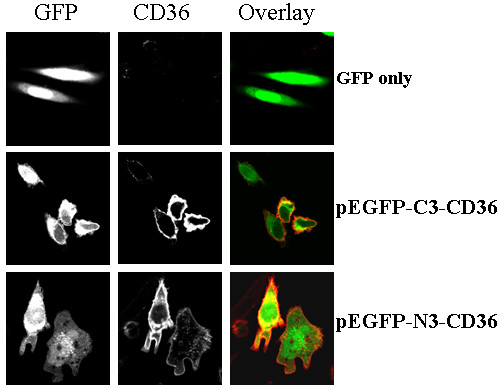
**Confocal microscopy to observe both GFP and huCD36 protein expression**. Constructs of pEGFP-C3-CD36, pEGFP-N3-CD36, and pEGFP-N3 vector alone, were transit-transfected into CHO cells for 48 hours, and the transfected cells were then immunostained with anti-CD36 antibody, counter-stained with TRIC-conjugated anti-mouse IgG secondary antibody. After washing several times, cells were mounted on slides and observed under confocal microscope. Top panel: pEGFP-N3 transfected alone; Middle panel: cells transfected with pEGFP-C3-CD36; Bottom panel: pEGFP-N3-CD36 transfected. Both GFP and CD36 were expressed in two constructs.

**Figure 3:**
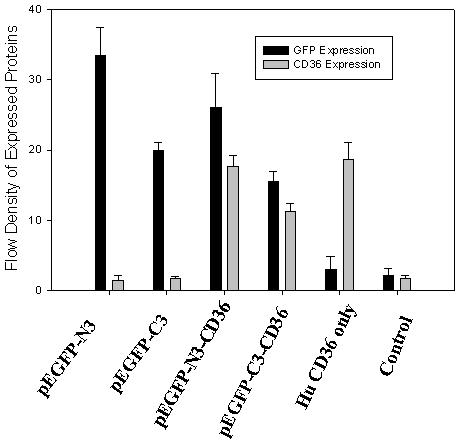
**Quantitative assays of GFP and CD36 expression**. The procedures of transfection were same as in Fig. 2. After transfection for 48 hours, cells were washed three times with PBS and then fixed with 4% paraformaldehyde for 30 min and then assayed under flow cytometry. The results are a summary of three independent experiments.

To verify if recombinant GFP-CD36 could be normally expressed in vivo, the fusion plasmids, the pEGFP-C3-CD36 and pEGFP-N3-CD36, were transfected into CHO cells with an ExGen 500 kit (Clontech). After 48 hours of incubation, transfected cells were immunofluorescently stained with anti-CD36 antibody, counter stained with TRIC-conjugated secondary antibody, and then either directly viewed under a fluorescent microscope or assayed by flow cytometery. As showing in Fig. 2, GFP were well expressed with either of the recombinant plasmids and the CD36 proteins were also normally expressed. Quantitative results by flow cytrometry demonstrated that CD36 proteins in pEGFP-N3-CD36 is about 30% stronger than that in pEGFP-C3-CD36 plasmids (Fig. 3).

### pEGFP-C3-CD36 limited OxLDL binding but not by pEGFP-N3-CD36

**Figure 4:**
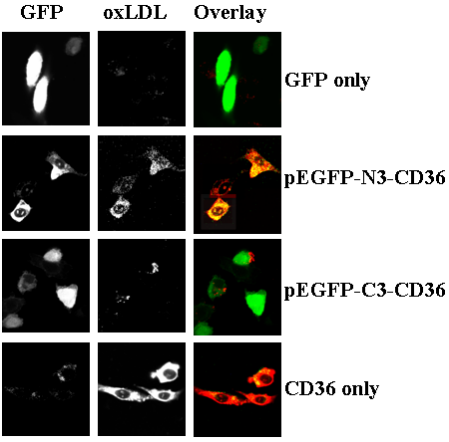
**pEGFP-C3-CD36 limited OxLDL uptake, but not for pEGFP-N3-CD36**. pEGFP-C3-CD36 and pEGFP-N3-CD36, as well as pEGFP-N3 alone were transit-transfected into CHO cells. After transfection for 48 hours, cells were rinsed twice with PBS and Dil-oxLDL at 10 μg/ml was incubated with the transfected cells for 1 hour. After rinsed for three times with PBS, cells were mounted on coverlids and observed under confocal microscope. Typical transfected and Dil-oxLDL was photographed. Dil-oxLDL was only weakly bound to pEGFP-C3-CD36 transfected cells.

As CD36 is a receptor for oxLDL, we wondered if, in its GFP-recombinant derivatives, the tag altered its affinity to binding oxLDL. To test this, CHO cells were transfected with either of the four plasmid DNAs, pEGFP-C3 and pEGFP-N3, pEGFP-C3-CD36, pEGFP-N3-Cd36, and pCDNA3-hCd36. After 48 hours of incubation, the transfected cells were assayed for Dil-oxLDL binding by uptaking. As shown in Fig. 4, for cells transfected with the control GFP plasmid, only a weak background of OxLDL was seen (Fig. 4, top panel); Cells transfected with pEGFP-N3-Cd36 showed a normal amount of dil-oxLDL binding (Fig. 4, second panel from the top). However, once transfected with peGFP-C3-CD36, only a very weak staining of Dil-oxLDL was seen (Fig. 4, the second panel from the bottom), which indicated that GFP tagged to different ends of CD36 functionally altered the property of binding OxLDL. Cells transfected with CD36 alone (pCDNA3-CD36) showed a strong Dil-oxLDL binding.

**Figure 5:**
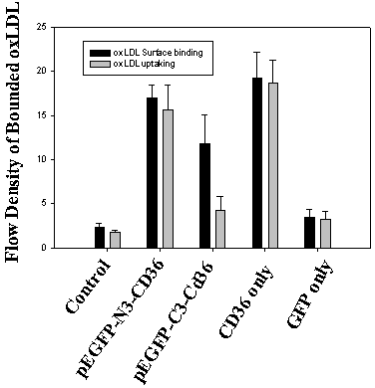
**Deficiency of uptake oxLDL by pEGFP-C3-CD36 was partially rescued by surface binding**. The transfection was same as described in Fig. 4. After 48 hours of transfection, cells were rinsed with PBS twice and fixed with 4% paraformaldehyde for 30 min, and then incubated with Dil-OxLDL at 10 μg/ml for 2 hours at room temperature. After washed for three times with PBS, cells were collected and assayed with flow cytometry. The results are an average of three experiments. Dark box: oxLDL uptaking and grey box: oxLDL surface binding.

Further, we hypothesized that the differences in Dil-oxLDL binding between the two ends of GFP-tagged cD36 are cytoplasmically affected by over-expressed GFP. If this is true, oxLDL should be relatively similar to those in surface binding. To the ends, transfected cells were first fixed with 4% formaldehyde and then incubated with Dil-oxLDL to lead to them sorely surface binding. As seen in Fig. 5, there no much change was seen on cells transfected with either pEGFP-N3-Cd36, pCDNA3-CD36, pEGFP-N3, controls in dil-oxLDL binding between two binding assay methods (Fig. 5). However, in pEGFP-C3-CD36 transfected cells, the amount of bound OxLDL in surface binding was increased to about 60%, compared to uptaking (Fig. 5, panel pEGFP-C3-CD36).

### Recombinant GFP-tagged CD36 doesn't change the property of pRBCs binding

**Figure 6:**
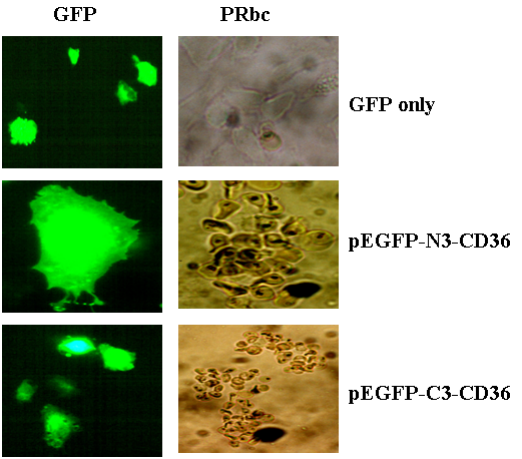
**Both N- and C-terminal tagged huCD36 bind normally to pRBCs**. After transfection with pEGFP-C3-CD36, pEGFP-N3-CD36 or pEGFP-N3 alone, cells were fixed with 4% paraformaldehyde for 20 min and then rinsed twice with PBS. The cells were then incubated with pRBCs for 4 hours at room temperature with gent shacking. pRBCs bindings were observed under fluorescent microscope and typical binding cells were photographed. pRBCs were bound to both pEGFP-C3-CD36 and pEGFP-N3-CD36 transfected cells.

As reported early, CD36 directly mediates cytoadherence of plasmodium falciparium parasitized erythrocytes [20]. We investigated whether recombinant GFPCD36 retains its property of pRBCs adherence. As shown in Fig. 6, cells transfected with either pEGFP-C3-CD36 or pEGFP-N3-CD36 displayed normal pRBCs adherence (Fig. 6, panels pEGFP-C3-CD36 and pEGFP-N3-CD36). However, no pRBCs binding was observed when pEGFP-C3 or pEGFP-N3 vector alone was transfected (Fig. 6, top panel).

## Discussion

In the present study, we first successfully created the fusion constructs of GFP to huCD36 at both the N- and C-termini, termed as pEGFP-C3-huCD36 and pEGFP-N3-CD36. Both constructs, as well as GFP-encoding plasmid and the CD36 cDNA clone, were normally expressed once they were transiently-transfected into CHO cells. By immunostaining with antiCD36 antibody, the results revealed that the CD36 proteins collectively existed in cellular membrane and GFPs, however, were concentrated in the cytoplasm (Fig. 2). Then, we assayed whether the fusion to the GFP protein could have influence on the physiological function of CD36 in the recombinant proteins. Firstly, we attempted to detect if co-expressed GFP proteins could modify Ox-LDL binding on CD36. Two kinds of binding assay methods, uptake and surface binding, were carried out. To our surprise, p-EGFP-N3-CD36 showed a normal binding to OxLDL in both of the binding assays. However, pEGFP-C3-CD36 showed almost no OxLDL binding in uptaking, and a much increased surface binding. Then we assayed for pRBC binding and the results confirmed that CD36 proteins expressed from both pEGFP-C3-CD36 and pEGFP-N3-CD36 showed normal adhesion to pRBCs (Fig. 6), and their bindings are similar to those as observes in normal CD36 expressing cells [20].

CD36 has been well defined as a membrane receptor that can function as a molecule with specificity for multiple ligands including collagen, pRBCs, TSP, apoptotic cells and modified lipoproteins [25,32,35,37]. Several earlier reports suggested that each ligand has a discrete non-contiguous region on CD36 molecule that may be spatially proximal. OxLDL bound to an immobilized fusion protein containing the amino-termini of p6–143 and p28–93 showed an approximately similar affinity as intact CD36 [26], which provided a conclusive evidence for oxLDL binding domain at P28–93 on CD36. However, the amino acid region at 93–120 was only for the TSP binding, but not for oxLDL binding [38,39]. In contrast, one report by using a chimeric CD36 construct with the murine sequence 155 – 183 substituting the equivalent human sequence suggested that the p155–183 domain may be critical for oxLDL binding [29]. Crandall et al (1999) reported that OxLDL competitively inhibits the adherence of pRBCs to CD36 but they suggest that, although oxLDL competitively inhibits the adherence of pRBCs, these ligand interact with distinct domains on the CD36 receptor [20]. Imach et al (2000) reported that the scavenger receptor class A, CD36, or CD68 can specifically recognize damaged apoptotic cells and they may play a major role in the clearance of apoptotic cells in the thymus, mediating the recognition and ingestion of apoptotic thymocytes [40]. All of the above reports seemed to come to a conclusion that CD36 embeds specific regions for binding of different ligands and some of them may co-interact. By sequence alignment of CD36 proteins from different species and other CD36 gene family members the N-terminal region from the amino acid 31–190 shows significant sequence conservation indicative of its functional importance [26], possibly in oxLDL binding.

The approach using recombinant fusion protein seemed to be a potent technique to detect specific amino acid domains and to analyze protein-protein interaction [26,30]. In our study, we used GFP-tagged to CD36 to investigate if the CD36 could be expressed in CHO cells and if GFP could affect CD36 function to interact with oxLDL and pRBCs. We choose to use GFP as fusion proteins based on the following reasons: The green fluorescent proteins (GFP), have been recently used for many cellular biology approaches to investigate target proteins' localization, physiological function and signal transduction, as well as gene expression [41,42]. Typically, proteins tagged to GFP were used to detect protein localization in living cells [43]. Also, GFP as a marker was used for gene expression [44], protein trafficking and secretion [45], and apoptosis assays [45,46]. Also GFP was extended to assay cellular physiological changes of enzyme activities and signal transduction [47,48]. In this study, our results observed that when CD36 attached to the C-terminal of GFP, binding of ox-LDL was deleted, but when CD36 was attached to the N-terminal, there is no much effect on ox-LDL binding. There are two possibilities that may cause this to happen: (1) the Ox-LDL binding domain is located at the N-terminal of CD36 and it closely contacts with GFP proteins, the expressed GFP proteins may directly affect oxLDL binding via protein-protein interaction, or change of protein conformation; (2) the expressed GFP proteins may directly or indirectly affected CD36 protein's physiological function. This finding indicated that preventing of atherosclerosis could be achieved via CD36 protein recombination.
